# Comparison of Autonomic Control of Blood Pressure During Standing and Artificial Gravity Induced via Short-Arm Human Centrifuge

**DOI:** 10.3389/fphys.2018.00712

**Published:** 2018-06-25

**Authors:** Ajay K. Verma, Da Xu, Michelle Bruner, Amanmeet Garg, Nandu Goswami, Andrew P. Blaber, Kouhyar Tavakolian

**Affiliations:** ^1^Department of Electrical Engineering, University of North Dakota, Grand Forks, ND, United States; ^2^Department of Biomedical Physiology and Kinesiology, Simon Fraser University, Burnaby, BC, Canada; ^3^Department of Engineering Science, Simon Fraser University, Burnaby, BC, Canada; ^4^Physiology Division, Otto Loewi Research Center for Vascular Biology, Immunology and Inflammation, Medical University of Graz, Graz, Austria

**Keywords:** microgravity, artificial gravity, blood pressure regulation, orthostatic intolerance, astronauts, short-arm human centrifuge, long duration spaceflight, cardiovascular deconditioning

## Abstract

Autonomic control of blood pressure is essential toward maintenance of cerebral perfusion during standing, failure of which could lead to fainting. Long-term exposure to microgravity deteriorates autonomic control of blood pressure. Consequently, astronauts experience orthostatic intolerance on their return to gravitational environment. Ground-based studies suggest sporadic training in artificial hypergravity can mitigate spaceflight deconditioning. In this regard, short-arm human centrifuge (SAHC), capable of creating artificial hypergravity of different g-loads, provides an auspicious training tool. Here, we compare autonomic control of blood pressure during centrifugation creating 1-g and 2-g at feet with standing in natural gravity. Continuous blood pressure was acquired simultaneously from 13 healthy participants during supine baseline, standing, supine recovery, centrifugation of 1-g, and 2-g, from which heart rate (RR) and systolic blood pressure (SBP) were derived. The autonomic blood pressure regulation was assessed via spectral analysis of RR and SBP, spontaneous baroreflex sensitivity, and non-linear heart rate and blood pressure causality (RR↔SBP). While majority of these blood pressure regulatory indices were significantly different (*p* < 0.05) during standing and 2-g centrifugation compared to baseline, no change (*p* > 0.05) was observed in the same indices during 2-g centrifugation compared to standing. The findings of the study highlight the capability of artificial gravity (2-g at feet) created via SAHC toward evoking blood pressure regulatory controls analogous to standing, therefore, a potential utility toward mitigating deleterious effects of microgravity on cardiovascular performance and minimizing post-flight orthostatic intolerance in astronauts.

## Introduction

Right from birth, humans experience gravity, which pulls the human body toward the earth with a force equivalent to the product of the body mass and the gravitational acceleration, i.e., 9.8 m/s^2^ (Antonutto and di Prampero, 2003). Therefore, quintessential physiological performance is highly dependent on gravity (Blaber et al., 2013b; Tanaka et al., 2016). Physiological adaptation, a result of long-term microgravity exposure, can cause changes in physiological functions (Lambertz et al., 2003; Hallgren et al., 2015; Otsuka et al., 2015; Morita et al., 2016; Harris et al., 2017). Of such, cardiovascular adaptation to microgravity can have detrimental effects on the autonomic control of blood pressure upon return to gravitational environment (Hargens et al., 2013; Tanaka et al., 2016). Orthostatic intolerance, an inability to regulate blood pressure on assuming upright stance (Robertson, 1999; Lambert and Lambert, 2014), is commonly experienced by astronauts on their return to Earth after long-duration spaceflight (Buckey et al., 1996; Lee et al., 2015). The success of envisioned Mars exploration would entail frequent long-duration spaceflight in the future (Manzey, 2004; Baisden et al., 2008; Clément et al., 2016). To this end, profound investigation of potential countermeasures is warranted to mitigate the adverse effects of microgravity on physiological performance to facilitate healthy life for astronauts on their return to Earth (Clément et al., 2016).

The transition of posture, from supine to standing, reduces the blood pressure at the brain level (∼70 mmHg) and increases the blood pressure at the feet level (∼200 mmHg), as a consequence of gravity (Hargens and Richardson, 2009; Hargens et al., 2013). Central blood volume, owing to gravity induced hydrostatic pressure gradient, is displaced below heart level and venous return and preload are reduced challenging blood pressure equilibrium. Regulating blood pressure is imperative for the sustainability of prolonged and stable upright stance. During physiological state of standing, blood pressure is regulated via reduced afferent discharge of the baroreceptor impulses localized in the carotid sinus and the aortic arch, leading to increased heart rate and systemic vascular resistance as a consequence of vagal withdrawal and sympathetic nerve activation (Smith et al., 1994; Heesch, 1999).

Therefore, to cope with the effects of orthostatic challenge on physiological performance, autonomic, baroreceptor, and vasomotor controls play a consequential role to various degrees. Enfeebled or impaired blood pressure regulatory controls owing to long-term microgravity exposure could lead to an abrupt decline in arterial blood pressure on assuming upright stance resulting in reduced cerebral perfusion (Olufsen et al., 2004, 2005; Shaw and Claydon, 2014). Thus, impaired autonomic control has an ominous effect on the individual’s ability to maintain standing (Antonutto and di Prampero, 2003; Hargens et al., 2013; Tanaka et al., 2016).

Ground-based experiments, performed to comprehend physiological response to microgravity simulated via bed rest immobilization (LeBlanc et al., 1988; Jeong et al., 2013; Norsk, 2014) and potential countermeasures such as exercise training, lower-body negative pressure, and artificial gravity (Goswami et al., 2008; Blaber et al., 2013a; Hargens et al., 2013), have concluded an intermittent exposure to artificial hypergravity as an important factor toward improving orthostatic tolerance (Evans et al., 2004; Stenger et al., 2007, 2012; Hargens et al., 2013). The short-arm human centrifuge (SAHC), in this regard, can serve as a promising training tool (Evans et al., 2004; Frett et al., 2014; Clément et al., 2015). The feasibility of short-arm centrifuge to be a part of a long duration spaceflight, owing to compact modern design, has opened new avenues toward minimizing the severity of microgravity-induced systemic deconditioning (Bukley et al., 2007; Zander et al., 2013; Frett et al., 2014; Diaz et al., 2015).

In the previous work from our group (Goswami et al., 2015a), we demonstrated the response of cardiovascular and the cerebrovascular system during 2-g centrifugation at feet to be analogous to orthostatic challenge exerted by standing in a natural gravity. However, the autonomic blood pressure regulatory controls during centrifuge induced artificial gravity in relation to standing in natural gravity remain to be generalized. In the current analysis, we extend our previous work to a comparison of the response of autonomic control of blood pressure during artificial gravity induced via SAHC in relation to standing. The autonomic control of blood pressure was assessed via causal heart rate-blood pressure interaction, spontaneous baroreflex sensitivity, and spectral analysis of SBP and RR time series.

## Materials and Methods

### Experimental Protocol and Data Acquisition

The detailed experimental protocol has been explained in the previous work from our group (Goswami et al., 2015a). Here, we briefly outline the experimental protocol with respect to the current research. All participants were pre-screened for physical and medical status. None of the participants had a prior history of cardiovascular, neurological, and musculoskeletal diseases or vasovagal syncope. Twelve hours prior to experimentation, all participants were required to refrain from alcohol, caffeine, and any medication.

In the centrifuge, the participant was strapped with their head near the center of 2.8-m radius centrifuge and feet outwards. The participant remained supine (Baseline) in the centrifuge for 20 min of baseline recording. After completion of baseline, the participant was transitioned with assistance into the standing position for 5 min (stand test). After the stand test, the participant lay supine (recovery) in the centrifuge for another 15 min, after which the centrifuge was ramped up to a rate that applied 1-g at feet (0.22 g at Middle Cerebral Artery and 0.39 g at heart) for 5 min and then increased to 2-g at feet (0.44 g at Middle Cerebral Artery and 0.75 g at the heart) for 5 min. Following 5 min of centrifugation at 2-g, the centrifuge rotation was slowed and halted in 30 s.

Data was acquired from 13 participants (age: 28.08 ± 8.4 year, height: 172 ± 6.9 cm, weight: 67.6 ± 10.5 kg, six females). The detailed demographic information is provided in **Table 1**. Continuous blood pressure was acquired from non-invasive finger photoplethysmography cuff (Portapress, FMS, Netherlands) using NI data acquisition (National Instruments, Inc., Austin, TX, United States) system at a sampling rate of 1000 Hz. Ethics approval for experimentation was obtained from the University of Toulouse. Experimentation complied with rules and regulations set forth by the research ethics board of the University of Toulouse. Written and informed consent form for participation was obtained from each participant prior to any experimentation.

**Table 1:** Detailed demographic information of study participants.

Participant #	Age	Height	Weight	Gender
1	23	172	73	Male
2	31	165	55	Female
3	24	164	56	Female
4	25	169	62	Female
5	25	176	75	Male
6	35	175	76	Male
7	23	165	53	Female
8	29	175	79	Male
9	24	174	55	Female
10	35	189	84	Male
11	39	178	73	Male
12	37	166	72	Male
13	32	169	68	Female

### Short-Arm Human Centrifuge

Short-arm human centrifuge is a training tool capable of creating artificial gravity of different g-load (Zander et al., 2013; Clément et al., 2015). Briefly, the head of participant is aligned close to the center of centrifuge rotation, while the feet are directed outwards from the center of rotation. In this orientation, the g-load is distributed linearly in a head-to-toe axis, i.e., the g-load at the feet is hypothesized to be proportional to the rotational speed of the centrifuge, while at the head the g-load is closer to zero. Although this does not simulate the typical hydrostatic difference created by standing in a natural gravity, it does generate similar physical stressors in the footward direction. The relation between artificial gravity created at feet on Earth and rotation of centrifuge can be described as; CF = $\frac{\text{rx}\omega^{2}}{\text{g}}$. Where CF is a centrifugal force, r is the radius at feet, ω is the rotational speed, and g is the Earths gravitational acceleration (Clément and Pavy-Le Traon, 2004). The centrifuge facility at Institute for Space Medicine and Physiology (MEDES), Toulouse, France was used in this research.

### Data Processing

Diastolic nadirs of each beat were first detected from the continuous blood pressure waveform to obtain diastolic blood pressure (DBP). The R-R time interval was obtained as the duration between two adjacent diastolic nadirs. Systolic blood pressure (SBP) was obtained as the maximum blood pressure between two adjacent diastolic nadirs. Beat-by-beat mean arterial pressure (MAP) was derived from blood pressure waveform as a mean value between two adjacent DBP locations. 5-min of data from each experimental condition was considered for analysis.

RR interval and SBP time series were interpolated using spline interpolation to generate an evenly sampled signal and resampled to 2 Hz with zero mean before conducting the spectral analysis. The Welch power spectral density (PSD) of RR and SBP was calculated in very low frequency (VLF, 0–0.04 Hz), low frequency (LF, 0.04–0.15), and high frequency (HF, 0.15–0.4 Hz) bands. There upon, the SBP power distributed (P) in the respective bands were normalized as VLF_nu_ = P_V LF_÷Total Power, LF_nu_ = P_LF_÷Total Power, and HF_nu_ = P_HF_÷Total Power, in case of RR signal the normalized power in only LF and HF frequency band was calculated as LF_nu_ = P_LF_÷(Total Power-P_V LF_) and HF_nu_ = P_HF_÷(Total Power-P_V LF_) where Total Power = P_V LF_+P_LF_+P_HF_ in accordance with recommendation in the literature (Malik et al., 1996). The PSD was computed with a Hamming window of size 128 samples and 50% overlap.

The arterial baroreflex sensitivity was calculated using sequence method (Bertinieri et al., 1988; Blaber et al., 1995) by using CardioSeries computer software V2.4^1^ similar to other research in the literature (Durand et al., 2015; Silva et al., 2015). Beat-to-beat RR intervals and SBP were input to the software, search for a sequence of at least three consecutive beats in which increase in SBP was followed by an increase in RR intervals (up slope) and decrease in SBP followed by a decrease in RR intervals (down slope) with a correlation greater than 0.8 was considered. The slope of linear regression between SBP and RR intervals was considered as a marker of spontaneous BRS.

The strength of closed loop heart rate-blood pressure interaction (RR↔SBP), signifying the feedforward (non-baroreflex) and feedback (baroreflex) controls of blood pressure was obtained using convergent cross mapping (CCM) similar to our previous work (Verma et al., 2017b; Xu et al., 2017). Prior to causality analysis, the evenly sampled continuous RR and SBP signals were resampled to 10 Hz. Mathematical details of the methodology are provided in the supplementary material of Sugihara et al. (2012) and in a book on time series analysis by Mccracken (2016).

### Statistical Analysis

Test for normal distribution of the mean of different variables studied in this research was conducted using Shapiro–Wilk test (SPSS, IBM Corporation, Armonk, NY, United States). A multiple comparison test was conducted using repeated measure of ANOVA (for normally distributed data) or Friedman test (data failed normality) followed by *post hoc* analysis using Tukey-HSD method to account for the significance of changes in the cardiovascular parameters and blood pressure regulatory indices during different experimental conditions. The test of significance was conducted using a statistical toolbox of MATLAB (The Mathworks, Inc., Natick, MA, United States). The test results at α = 0.05 were considered significant. All tabular data in the article are presented as mean ± SD unless mentioned otherwise.

## Results

**FIGURE 1:**
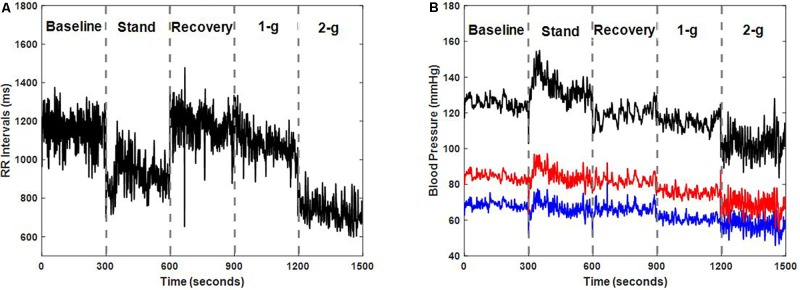
Dynamics of RR intervals **(A)** and blood pressure **(B)** i.e., systolic blood pressure (SBP) (black), diastolic blood pressure (DBP) (blue), and mean arterial pressure (MAP) (red) in response to different experimental conditions for one participant (male, age: 35 years, height: 175 cm, weight: 76 kg).

Test of normality resulted in data exhibiting mixed behavior given the limited sample size, therefore, Friedman test followed by *post hoc* analysis using Tukey-HSD method was conducted to account for difference exerted by different experimental conditions on the cardiovascular parameters as well as on the blood pressure regulatory indices. **Table 2** summarizes the behavior of cardiovascular parameters during baseline, standing, recovery, centrifugation of 1-g, and 2-g. Stand test or application of centrifugation inflicted no change in SBP (*p* = 0.13), DBP (*p* = 0.27), or MAP (*p* = 0.28). RR intervals reduced significantly during standing (*p* < 0.001) and 2-g (*p* < 0.001) compared to supine. Additionally, RR intervals reduced significantly during standing (*p* < 0.001) and 2-g (*p* < 0.001) compared to recovery as well as during 2-g (*p* = 0.02) compared to 1-g. No change in RR intervals was obtained during recovery (*p* = 0.99) and 1-g (*p* = 0.09) compared to baseline. Furthermore, no change (*p* = 0.57) in RR intervals was observed between standing and 2-g. **Figure 1** shows an example of RR intervals and blood pressure dynamics in response to different experimental conditions.

**Table 2:** Values (mean ± SD) of cardiovascular parameters during different experimental conditions.

Parameters	Baseline	Stand	Recovery	1-g	2-g
R-R (ms)	967 ± 177	754 ± 135^†^	957 ± 155	879 ± 158	669 ± 115^†‡^
SBP (mmHg)	110 ± 15	122 ± 21	118 ± 13	119 ± 16	114 ± 21
DBP (mmHg)	58 ± 9	65 ± 17	62 ± 10	61 ± 12	61 ± 16
MAP (mmHg)	74 ± 11	80 ± 18	78 ± 12	76 ± 12	74 ± 17

*Results were considered significant at α = 0.05. ^∗^Represents significant difference from baseline. ^†^Represents significant difference from recovery. ^‡^Represents significant difference from 1-g*.

**FIGURE 2:**
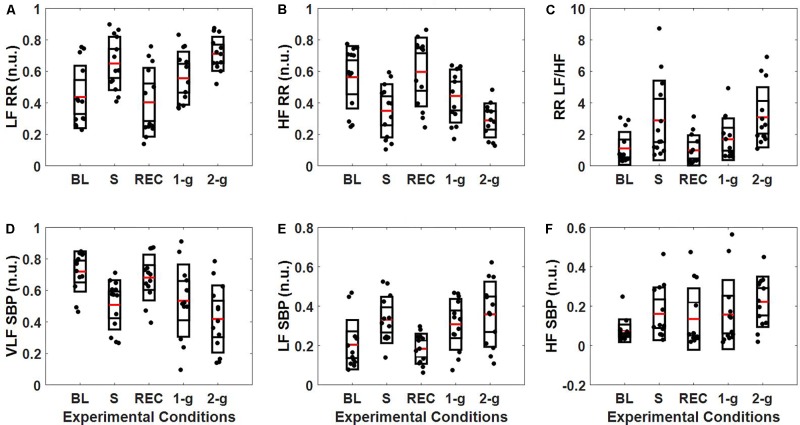
Distribution of SBP and RR intervals spectral power (n.u.). The figure details RR **(A–C)** and SBP **(D–F)** spectral power distribution in the VLF (0–0.04 Hz), LF (0.04–0.15 Hz, and HF (0.15–0.4 Hz) bands during baseline (BL), stand (S), recovery (REC), 1-g, and 2-g experimental protocol.

**Figure 2** summarizes the normalized spectral power distribution in the VLF, LF, and HF bands of SBP and LF, HF, and LF/HF ratio of RR intervals. The spectral power distribution in the respective frequency bands of SBP and RR in absolute and normalized units is summarized in **Table 3**. **Table 4** lists the *post hoc* comparison *p*-value between experimental conditions for respective frequency bands. Significant change was observed in low-frequency SBP power both in normalized and absolute units during standing (*p* = 0.04 and *p* = 0.01) and 2-g (*p* < 0.001 and *p* < 0.001) compared to baseline. Moreover, the low-frequency SBP power (both in normalized and absolute power) was significantly different during standing and 2-g compared to recovery. In addition, the SBP_LF_ and SBP_HF_ in absolute power were observed to be significantly different (*p* < 0.05) at 2-g compared to 1-g (**Table 3**). No difference (*p* > 0.10) was the behavior of such variable at 2-g compared to standing (**Table 4**).

**Table 3:** Values (mean ± SD) of blood pressure regulatory indices in response to different experimental conditions.

Blood pressure regulatory indices	Baseline	Stand	Recovery	1-g	2-g
SBP_V LF_ (n.u.)	0.72 ± 0.13	0.51 ± 0.16	0.68 ± 0.15	0.54 ± 0.23	0.42 ± 0.21^†^
SBP_LF_ (n.u.)	0.20 ± 0.13	0.33 ± 0.12^†^	0.18 ± 0.08	0.31 ± 0.11	0.35 ± 0.17^†^
SBP_HF_ (n.u.)	0.08 ± 0.06	0.16 ± 0.13	0.13 ± 0.16	0.16 ± 0.18	0.22 ± 0.13
SBP_V LF_ (mmHg^2^)	22.02 ± 11.58	21.60 ± 11.78	22.43 ± 20.63	16.07 ± 16.14	29.67 ± 32.84
SBP_LF_ (mmHg^2^)	5.80 ± 3.95	14.36 ± 8.44^†^	5.19 ± 4.05	6.91 ± 4.86	20.93 ± 13.02^†‡^
SBP_HF_ (mmHg^2^)	1.89 ± 1.01	6.41 ± 6.49	1.94 ± 1.36	2.37 ± 1.97	11.41 ± 8.44^†‡^
RR_LF_ (n.u.)	0.44 ± 0.20	0.65 ± 0.17^†^	0.40 ± 0.22	0.56 ± 0.17	0.71 ± 0.11^†^
RR_HF_ (n.u.)	0.56 ± 0.20	0.35 ± 0.17^†^	0.60 ± 0.22	0.44 ± 0.17	0.29 ± 0.11^†^
RR_LF/HF_	1.11 ± 1.04	2.89 ± 2.53^†^	0.99 ± 0.96	1.69 ± 1.33	3.08 ± 1.91^†^
RR_LF_ (second^2^)	1.45 ± 1.40	1.25 ± 1.31	1.25 ± 1.42	1.50 ± 2.06	1.23 ± 1.09
RR_HF_ (second^2^)	2.53 ± 3.14	1.01 ± 1.89^†^	3.78 ± 6.43	1.88 ± 3.53	0.51 ± 0.60^†^
BRS_upslope_ (ms/mmHg)	31.18 ± 20.19	10.40 ± 4.78^†^	30.65 ± 20.91	20.10 ± 9.43	7.52 ± 3.38^†‡^
BRS_downslope_ (ms/mmHg)	29.31 ± 18.15	10.85 ± 6.39^†^	27.51 ± 16.78	21.32 ± 10.16	6.80 ± 2.67^†‡^
RR→SBP	0.95 ± 0.03	0.93 ± 0.03	0.93 ± 0.03	0.92 ± 0.05	0.92 ± 0.04
SBP→RR	0.83 ± 0.09	0.91 ± 0.03*	0.88 ± 0.07	0.91 ± 0.04*	0.89 ± 0.05

*The results were considered significant at α = 0.05. ^∗^Represents significant difference from baseline. ^†^Represents significant difference from recovery. ^‡^Represents significant difference from 1-g.*

**Table 4:** Comparison of changes in blood pressure regulatory indices inflicted by different experimental conditions.

Variables and conditions to compare	Baseline vs. stand	Baseline vs. recovery	Baseline vs. 1-g	Baseline vs. 2-g	Stand vs. 1-g	Stand vs. 2-g
SBP_V LF_ (n.u.)	0.07	0.99	0.17	0.001^a^	0.99	0.79
SBP_LF_ (n.u.)	0.04b	0.99	0.09	<0.001^a^	0.99	0.79
SBP_LF_ (abs power)	0.01a	0.99	0.90	<0.001^a^	0.12	0.79
SBP_HF_ (abs power)	0.09	0.99	0.98	<0.001^a^	0.27	0.41
RR_LF_ (n.u.)	0.13	0.79	0.85	0.007^a^	0.65	0.85
RR_HF_ (n.u.)	0.12	0.79	0.85	0.007^a^	0.65	0.85
RR_LF/HF_	0.12	0.79	0.85	0.007^a^	0.65	0.85
RR_HF_ (abs power)	0.02b	0.99	0.12	0.001^a^	0.97	0.90
BRS_upslope_	0.007a	0.99	0.41	<0.001^a^	0.48	0.79
BRS_downslope_	0.003a	0.99	0.65	<0.001^a^	0.16	0.72
SBP→RR	0.007a	0.21	0.04^b^	0.16	0.98	0.79

*Table lists post-hoc comparison p-values. Only variables showing significant Friedman test results are listed. ^a^Represents significant result at α = 0.01. ^*b*^Represents significant result at α = 0.05*.

The spectral power (n.u.) in the LF and HF frequency bands of RR intervals increased (*p* = 0.007) and decreased (*p* = 0.007) significantly only at 2-g compared to baseline (**Table 4**). The LF/HF ratio increased significantly at 2-g (*p* = 0.007) compared to baseline. Moreover, the three metrics were significantly different (*p* < 0.05) during standing and 2-g compared to recovery (**Table 3**). In terms of absolute power no change (*p* = 0.87) was observed in the RR_LF_, while RR_HF_ decreased significantly (*p* < 0.05) during standing and 2-g compared to baseline and recovery (**Table 3**). No difference (*p* > 0.10) in the dynamics of RR spectral power was obtained at 2-g compared to standing (**Table 4**).

**FIGURE 3:**
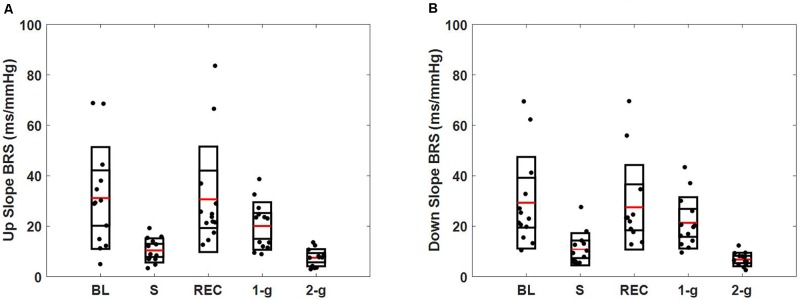
Spontaneous baroreflex sensitivity determined via sequence method. Figure details the distribution of up slope BRS **(A)**, down slope BRS **(B)** during baseline (BL), stand (S), recovery (REC), 1-g, and 2-g experimental protocol.

Baroreflex sensitivity decreased (both up and down slope) significantly during standing (up slope, *p* = 0.007 and down slope, *p* = 0.003) and 2-g (up slope, *p* < 0.001 and down slope, *p* < 0.001) compared to baseline. Significant decline in BRS was observed during standing (up slope, *p* = 0.003 and down slope, *p* = 0.004) and 2-g (up slope, *p* < 0.001 and down slope, *p* < 0.001) compared to recovery as well as at 2-g (up slope, *p* = 0.04 and down slope *p* = 0.005) compared to 1-g (**Table 3**). No change (up slope, *p* = 0.79 and down slope, *p* = 0.72) in BRS was observed at 2-g compared to standing (**Table 4**). The distribution of up slope and down slope BRS for the study participants is detailed in **Figure 3**.

**FIGURE 4:**
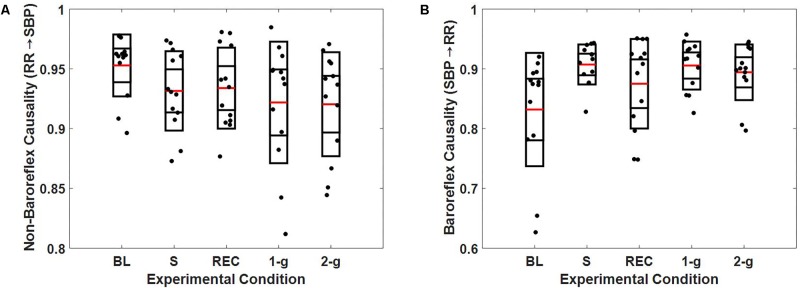
Boxplot representation of non-baroreflex **(A)** and baroreflex **(B)** causalities in response to baseline (BL), stand (S), recovery (REC), 1-g, and 2-g experimental protocol.

The optimal embedding dimension to perform non-linear state space reconstruction in CCM was determined via false nearest neighbor algorithm at a delay of 10 samples to account for changes within a heartbeat range. The optimal dimension of reconstruction was determined to be 4 for SBP and RR based on the minimization of false nearest neighbor using CRP toolbox in MATLAB (Kennel and Brown, 1992; Marwan, 2017). Therefore, the RR↔SBP causality was computed at an embedding dimension of 4 and delay of 10 samples unless mentioned otherwise. The causal behavior between RR and SBP is detailed in **Figure 4**.

At supine rest, the non-baroreflex arm of the heart rate and blood pressure interaction was significantly higher than the baroreflex arm. During standing (*p* = 0.007) and 1-g centrifugation (*p* = 0.04), a significant increase in baroreflex arm of the interaction compared to supine was observed, while no change (*p* = 0.26) in the non-baroreflex arm of the interaction was observed during experimental conditions. No change (*p* = 0.16) was observed in the baroreflex arm of the interaction at 2-g compared to baseline. Additionally, no difference between the standing and 2-g centrifugation was observed in the baroreflex arm (SBP→RR, *p* = 0.79 of the heart rate and blood pressure interaction (**Table 3**).

## Discussion

The current research investigated the capability of centrifugation (1-g and 2-g) at feet to evoke autonomic control of blood pressure analogous to standing. The autonomic blood pressure regulation mechanisms via causal heart rate-blood pressure interaction, spontaneous baroreflex sensitivity, and spectral analysis of SBP and RR were studied. The analysis results ascertained previously contemplated yet undocumented potential of SAHC to evoke autonomic blood pressure control analogous to standing in natural gravity, therefore, a potential utility toward minimizing the adverse effects of long-term microgravity exposure on the cardiovascular performance, hence, minimizing orthostatic intolerance in astronauts upon return to Earth.

The success of future inter-planetary missions to Mars depends on the design of pertinent countermeasures to mitigate the adverse effects of spaceflight deconditioning. The shift of central blood volume above thoracic leading to increased ventricular filling, stroke volume, and cerebral blood flow is an immediate consequence of physiological adaptation to microgravity. Accordingly, baroreceptor unloading, autonomic sympathetic nerve activity, and the vasomotor control remain vastly inhibited for the duration of spaceflight (White and Averner, 2001; Hargens and Richardson, 2009; Williams et al., 2016). The prolong inhibition of such blood pressure regulatory controls can have an adverse effect on individual’s orthostatic tolerance level, which can be analogous to aging and/or pathology (Takamatsu et al., 2016; Goswami, 2017).

Furthermore, decreased blood flow to the peripheral regions, especially to the calf skeletal muscles, render posture muscle group with nutritional scarcity, and is a major contributor toward skeletal muscle atrophy, bone remodeling, and decline in the calf circumference (LeBlanc et al., 1988; Lambertz et al., 2003; Stewart et al., 2004). While assuming upright stance on return to the gravitational environment, the blood pressure regulatory controls such as autonomic blood pressure controls and skeletal muscle pump remains vastly ineffective. Such change in physiological function can lead to excessive pooling of central blood volume in the lower periphery, resulting in cerebral perfusion reduction leading to feeling of dizziness and potentially syncope.

Thus, an external system capable of evoking autonomic control of blood pressure (baroreceptor unloading leading to increased sympathetic, decreased vagal activity, and increased systemic vascular resistance) and simultaneously increasing blood flow to the calf musculature to assure adequate nutritional and metabolic supply can mitigate the deterioration of cardiovascular performance associated with long-term exposure to microgravity.

Exercise training and lower-body negative pressurization have been utilized as a potential countermeasure to spaceflight deconditioning. However, traditionally used aerobic exercise, resistance training, and lower-body suction has been limited in effect due to their inability to challenge multiple physiological systems that are associated with standing (Bukley et al., 2007; Clément et al., 2015; Artiles et al., 2016). Consequently, interest has shifted toward SAHC as a training tool to minimize microgravity-induced physiological deconditioning (Clément and Pavy-Le Traon, 2004). SAHC, given its capability to create artificial gravity and has a potential to produce hydrostatic gradients analogous to standing and evoke multiple physiological systems simultaneously (Zander et al., 2013; Diaz et al., 2015). Achieving desired performance from SAHC, however, is contingent on an ideal choice of g-load. High g-load could initiate early syncopal symptoms, while low g-load could be insufficient to induce strenuous perturbation to hemodynamic homeostasis, and therefore, fails to evoke desired autonomic control of blood pressure. The autonomic control of blood pressure via the conventional approach of arterial baroreflex sensitivity and heart rate variability have been shown to exhibit microgravity or hypergravity induced alteration in the autonomic performance (Yanagida et al., 2014; Fontolliet et al., 2015). In this research, we investigated the capability of 1-g and 2-g centrifugation toward evoking autonomic controls of blood pressure. Previous research have demonstrated 2-g to be under the safe limit in addition to being strenuous enough to evoke physiological responses analogous to standing (Iwasaki et al., 2001; Goswami et al., 2015a).

We applied external perturbation to the hemodynamic homeostasis via stand test and centrifugation of 1-g and 2-g at feet. A recovery period followed a stand test to minimize its influence on the autonomic behavior during centrifugation. The comparison result of baseline and recovery period is summarized in **Tables 1, 4**. The results suggest the dynamics of studied variables were not different between the two conditions, therefore, the residual effects of standing was minimized and not likely to influence the autonomic behavior during centrifugation. The experimental conditions inflicted no change in SBP (*p* = 0.13), DBP (*p* = 0.27), or MAP (*p* = 0.28). This observation suggests that blood pressure was well-regulated during orthostatic challenge evoked via standing and centrifugation by active autonomic control of blood pressure. A significant decline was observed in the power distributed in the HF band of RR during standing (*p* = 0.02) and 2-g (*p* = 0.001) compared to baseline (**Table 4**), suggesting withdrawal of vagal nerve activity; resulting in a shift of sympatho-vagal balance toward sympathetic activity. Also, increase in LF SBP power was observed during standing and 2-g compared to supine (**Table 3**). Additionally, baroreflex sensitivity (both up slope and down slope) declined during standing and 2-g compared to supine (**Table 3**), which is the result of decreased vagal activity (**Table 4**) and increased heart rate or reduced RR intervals (**Table 1**) contributing toward maintenance of blood pressure equilibrium. While these markers of autonomic controls of blood pressure were significantly different from baseline during standing and 2-g, no change (*p* > 0.10) in the autonomic blood pressure control between standing and 2-g was observed (**Table 4**). Moreover, no change (**Table 4**) was observed in these indices between baseline and 1-g. This observation suggests 2-g is more closely related to standing compared to 1-g in terms of stimulating autonomic blood pressure regulatory controls.

While the spectral analysis of SBP and RR time series and baroreflex sensitivity are well-accepted norm to account for the autonomic control of blood pressure, criticism of such approaches in the literature is also prevalent for the inability of the spectral method to account for the non-linearity of underlying physiology and BRS for not able to address the closed loop heart rate and blood pressure interaction (Blaber et al., 1995; Malik et al., 1996; Zhong et al., 2006; Svacinova et al., 2015; Silvani et al., 2017). As such, in addition to traditional measures, the current article studied the non-linear causal heart rate and blood pressure interaction, a closed loop control system. Where the feedforward control signifies the Frank-Starling effect on blood pressure while the feedback control accentuates the baroreflex control of blood pressure. We studied the strength of feedforward (non-baroreflex, RR→SBP) and feedback (baroreflex, SBP→RR) controls of blood pressure during different experimental conditions (Faes et al., 2013; Javorka et al., 2017).

The results of closed loop heart rate and blood pressure interaction are detailed in **Figure 4**. No change (*p* > 0.05) was observed in the dynamics of non-baroreflex (RR→SBP) causality during standing or 2-g compared to supine. However, a significant increase was observed in the baroreflex (SBP→RR) causality; both during standing (*p* = 0.009) and 1-g (*p* = 0.04), **Table 4**. No change (*p* = 0.16) was observed in SBP→RR causality during 2-g compared to baseline. The slight decline in baroreflex causality at 2-g compared to 1-g (**Table 3**) could be due to the fact that 2-g was more stressful than standing and 1-g evident from certain study variables [SBP_V LF_, SBP_HF_ (n.u.), RR_HF_ (n.u.), and RR_LF_ (n.u.), **Table 4**], which changed only at 2-g compared to baseline. Furthermore, heart rate, BRS, SBP_LF_, and SBP_HF_ changed significantly at 2-g compared to 1-g (**Table 3**), which further hints toward 2-g being more stressful than 1-g. The RR intervals reduced at 1-g (*p* = 0.09) albeit not significantly but given limited sample size it does hint toward 1-g being more challenging than baseline. The baroreflex coupling is observed to decline before syncope (Faes et al., 2006), in our study syncope was not evident in the study participants, however, a decline in baroreflex causality indicates if exposed to 2-g centrifugation for an extended period, observation of syncopal symptom is plausible. Additionally, no change was observed in the non-baroreflex (*p* = 0.80) or baroreflex (*p* = 0.88) causal events during centrifugation compared to standing (**Table 3**). Therefore, the observations of current study suggest centrifugation at feet is capable of evoking autonomic control of blood pressure analogous to standing.

### Limitations and Future Directions

Experimental protocol in the current study was not randomized, i.e., centrifugation always followed the stand test. Although a 15-min recovery period was adopted to minimize the effects of stand test on centrifugation, in future study randomized experimental protocol should be considered. Furthermore, due to the unavailability of the respiration signal, the role of hypergravity toward the dynamics of respiration could not be studied. Respiration is known to affect both heart rate (RR) as well as blood pressure (Faes et al., 2011; Porta et al., 2012). Additionally, it may also play a role toward facilitating venous return via the physiology of respiration pump (Miller et al., 2005). Therefore, the role of respiration toward facilitating blood pressure homeostasis in response to orthostatic challenge shall be investigated in the future. Moreover, the blood volume redistribution in the splanchnic bed and the lower periphery due to standing and 2-g centrifugation shall also be measured and compared in the future. Orthostatic challenges evoked via a source that eliminates the effect of gravity such as lower-body negative pressure is observed to be different from that due to natural gravity (such as head-up tilt) (Taneja et al., 2007). Certain blood pressure regulatory controls changed only at 2-g compared to supine (**Table 4**), which indicate 2-g was more stressful than standing. Accurate information regarding the degree of blood pooling achieved during each experimental condition will shed further light pertaining to the vigor of 2-g in relation to standing.

Moreover, additional mechanisms that account for blood pressure regulation such as skeletal muscle pump (cardio-postural blood pressure regulation) shall also be investigated and compared in the future (Garg et al., 2014; Verma et al., 2017a; Xu et al., 2017). Furthermore, due to small sample size, the gender effect on blood pressure regulation and alteration in the dynamics of such behavior under artificial hypergravity remains to be understood. Female astronauts account for approximately 22% of total astronaut population (Harm et al., 2001), and studies have demonstrated significant gender difference in autonomic mechanisms leading to stable stance and in response to countermeasures designed to mitigate deleterious effect of spaceflight deconditioning (Arzeno et al., 2013; Goswami et al., 2015b; Hughson et al., 2016; Macaulay et al., 2016). Therefore, generalization of gender effect would further improve our understanding regarding the potential of SAHC as a training tool toward evoking blood pressure regulatory controls analogous to standing.

## Conclusion

Cardiovascular adaptation to microgravity impairs autonomic control of blood pressure, consequently, astronauts are susceptible to orthostatic intolerance on return to gravitational environment. Sporadic training in artificial hypergravity is proposed to mitigate the effects of spaceflight deconditioning. SAHC is a promising tool for simulating artificial gravity of different g-loads. The response of blood pressure regulatory controls to simulated hypergravity in relation to standing is not well-established in the literature.

In the current article, we investigated the response of autonomic control of blood pressure during centrifugation (1-g and 2-g) in relation to standing. While no difference was observed in the autonomic control of blood pressure between standing and centrifugation, the blood pressure regulatory indices during standing and centrifugation (mostly 2-g) were significantly different from baseline (**Table 4**). The findings of the current study lead us to conclude that 2-g centrifugation at feet via SAHC has potential to evoke autonomic control of blood pressure analogous to standing, therefore, a potential training tool toward reducing orthostatic intolerance in astronauts on their return to Earth.

## Author Contributions

NG and AB conceived the centrifuge study and designed the experiment. AV and KT conceived the data analysis steps. MB, NG, and AB acquired the data. DX preprocessed the data. AV performed the data and statistical analyses, wrote the manuscript, and created the figures and tables. AV, DX, AG, NG, AB, and KT interpreted the results. All authors contributed to manuscript editing and approved the final version for publication.

## Conflict of Interest Statement

The authors declare that the research was conducted in the absence of any commercial or financial relationships that could be construed as a potential conflict of interest.

## Footnotes

1 http://www.danielpenteado.com
